# Case Report: Primary gastric extraskeletal osteosarcoma and literature review

**DOI:** 10.3389/fmed.2026.1901151

**Published:** 2026-08-19

**Authors:** Xiangrui Meng, Zheng Ma, Zihe Dong, Jie Cao, Yanyan Lin

**Affiliations:** 1 The First Clinical Medical School, Lanzhou University, Lanzhou, Gansu, China; 2 Department of General Surgery, The First Hospital of Lanzhou University, Lanzhou, Gansu, China; 3 Department of Clinical Laboratory, The First Hospital of Lanzhou University, Lanzhou, Gansu, China

**Keywords:** case report, diagnosis, extraskeletal osteosarcoma, gastric tumor, treatment

## Abstract

Extraskeletal osteosarcoma is a rare and aggressive malignant mesenchymal tumor that accounts for approximately 1% of all soft tissue sarcomas and usually arises in the deep soft tissues of the extremities. Primary visceral involvement is extremely uncommon. We report a rare case of primary gastric extraskeletal osteosarcoma in a 65-years-old woman who presented with progressive abdominal pain, distension, nausea, vomiting, and cessation of flatus and defecation. Contrast-enhanced abdominal computed tomography revealed a large cystic-solid mass in the left middle and lower abdomen, with linear calcifications and central non-enhancing areas suggestive of necrosis and cystic degeneration. Because of worsening obstructive symptoms, an emergency radical distal gastrectomy was performed. Histopathological examination showed spindle-shaped tumor cells arranged in sheets, with multifocal lace-like malignant osteoid formation. Immunohistochemical findings, together with exclusion of metastatic skeletal osteosarcoma, supported the diagnosis of primary gastric extraskeletal osteosarcoma. Adjuvant chemotherapy was recommended after surgery, but was declined because of the patient’s poor general condition. No evidence of local recurrence or distant metastasis was observed during 9 months of follow-up. This case highlights that primary gastric extraskeletal osteosarcoma, although extremely rare, should be considered in the differential diagnosis of atypical gastric or intra-abdominal masses with calcification, necrosis, and spindle-cell morphology. Complete surgical resection remains the main treatment, and close long-term surveillance is essential because of the high risk of recurrence and metastasis.

## 1 Introduction

Osteosarcoma is one of the most common high-grade malignant tumors of the skeletal system and is characterized by the direct production of osteoid matrix or immature bone by malignant tumor cells. In rare cases, osteosarcoma may arise outside the skeletal system; this entity is known as extraskeletal osteosarcoma (EOS). EOS is a rare and highly aggressive malignant mesenchymal tumor that produces osteoid, bone, or chondroid material without direct attachment to bone or periosteum (1). It accounts for approximately 1% of all soft tissue sarcomas and 4.3% of all osteosarcomas (2). EOS predominantly affects adults in their fourth to sixth decades of life and shows a slight male predominance. The most common anatomical sites are the deep soft tissues of the thigh, buttock, and shoulder, whereas primary visceral involvement is uncommon (3). The etiology of EOS remains largely unclear, although previous studies have suggested radiation exposure as a potential risk factor (4, 5). Diagnosis is challenging due to the disease’s rarity and the non-specific clinical and radiological manifestations. Therefore, a definitive diagnosis relies on careful histopathological evaluation, adequate tumor sampling, identification of malignant osteoid formation, and appropriate immunohistochemical studies to exclude other differential diagnoses. Regarding treatment, multimodal strategies centered on complete surgical resection have been used for EOS; however, the overall prognosis remains poor, with high rates of local recurrence and distant metastasis (6–8).

Because of its extreme rarity, diagnostic complexity, aggressive clinical behavior, and the absence of well-established evidence-based treatment guidelines, each confirmed case of primary EOS deserves detailed documentation. Such reports may help improve understanding of this rare malignancy and provide useful clinical evidence for future diagnosis and management. Herein, we report a pathologically confirmed case of primary gastric EOS, focusing on its clinical presentation, imaging findings, histopathological and immunohistochemical features, treatment, and follow-up. This case may broaden the current understanding of the anatomical spectrum of EOS and highlight the importance of considering rare mesenchymal malignancies in the differential diagnosis of atypical gastric tumors.

## 2 Case description

**FIGURE 1:**
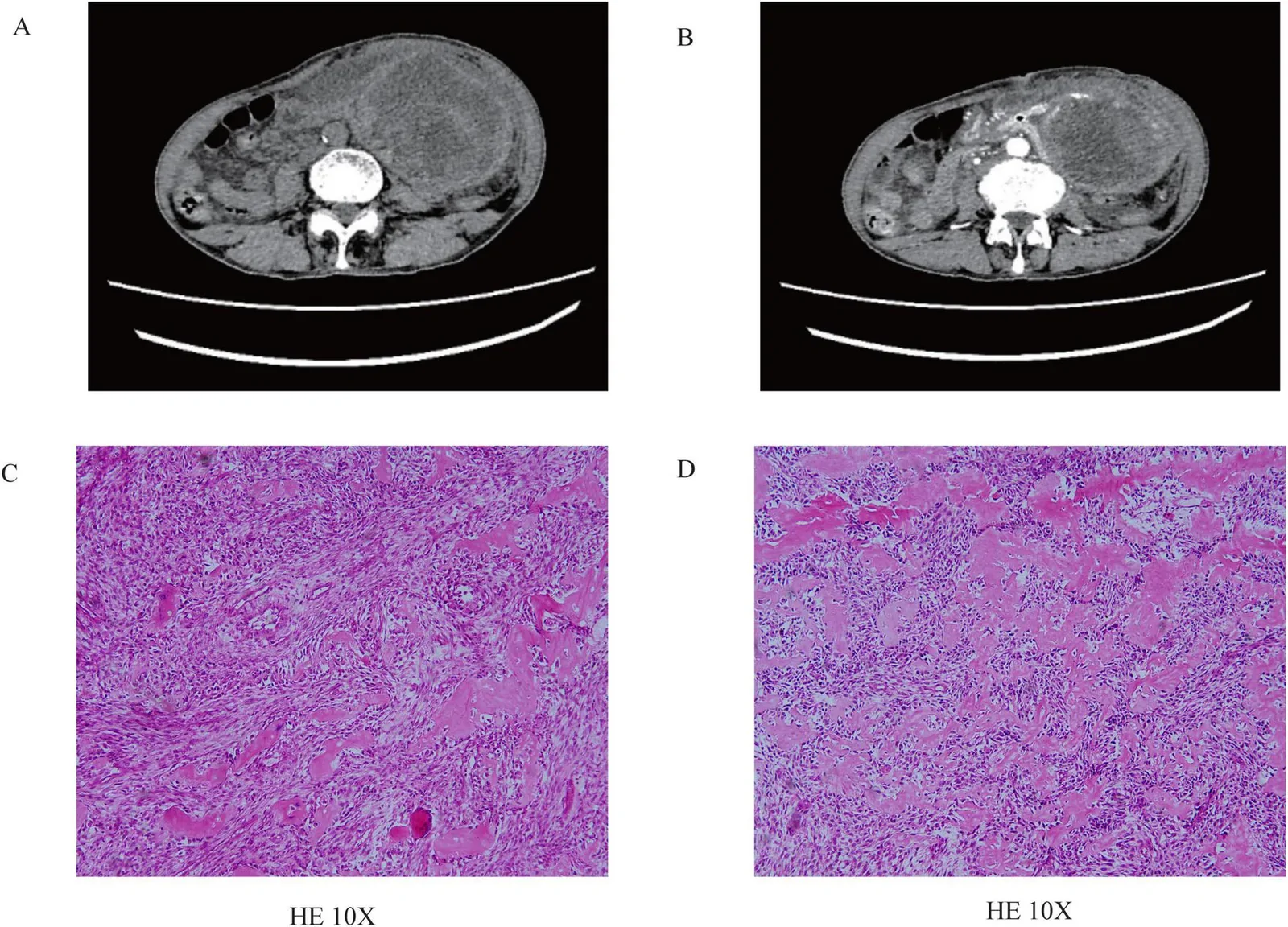
Radiological and histopathological findings. **(A,B)** Contrast-enhanced abdominal CT showed a large cystic-solid mass with heterogeneous density, linear calcifications, central necrosis, and cystic degeneration. **(C,D)** Hematoxylin and eosin staining showed spindle-shaped tumor cells arranged in sheets, with multifocal lace-like irregular osteoid formation.

A 65-years-old woman was admitted to our hospital with progressive abdominal pain and distension, accompanied by cessation of flatus and defecation for 3 days. One week before admission, she developed intermittent dull periumbilical pain with abdominal distension but did not seek medical attention. Three days before admission, the pain became persistent and sharp, with progressive aggravation of abdominal distension, complete cessation of flatus and defecation, and frequent nausea and vomiting. She had no history of fever, melena, or hematemesis. She denied any previous abdominal radiotherapy or trauma. Physical examination revealed abdominal guarding, mild tenderness below the xiphoid process, and marked tenderness with rebound pain in the left lower abdomen. A firm, fixed, and tender mass was palpable in the left lower abdomen. Bowel sounds were markedly decreased. Biochemical tests revealed a total bilirubin level of 22.96 μmol/L and a direct bilirubin level of 10.32 μmol/L, with other parameters unremarkable, and the only elevated tumor marker was carbohydrate antigen 125 at 122.7 U/ml. Contrast-enhanced abdominal computed tomography showed a cystic-solid mass in the left middle and lower abdomen, measuring approximately 93.5 mm × 115.8 mm × 118.2 mm. The lesion showed heterogeneous density, with linear calcifications in the wall and patchy non-enhancing areas in the central region, suggestive of necrosis and cystic degeneration. On enhanced images, the peripheral solid components showed moderate delayed enhancement. The boundary between the lesion and the adjacent small intestine or mesentery was indistinct. The greater curvature of the gastric body and adjacent small bowel loops were compressed. Dilatation and fluid accumulation were observed in bowel loops near the margin of the lesion. A large amount of abdominopelvic fluid was present, with partial high-density components and blurred abdominal fat planes. Multiple enlarged lymph nodes were seen in the hepatogastric space and retroperitoneum, with the largest measuring approximately 11 mm in short-axis diameter and showing homogeneous enhancement (Figures 1A, B). Chest computed tomography revealed bilateral pulmonary nodular and patchy lesions, which were considered suggestive of pulmonary tuberculosis.

**FIGURE 2:**
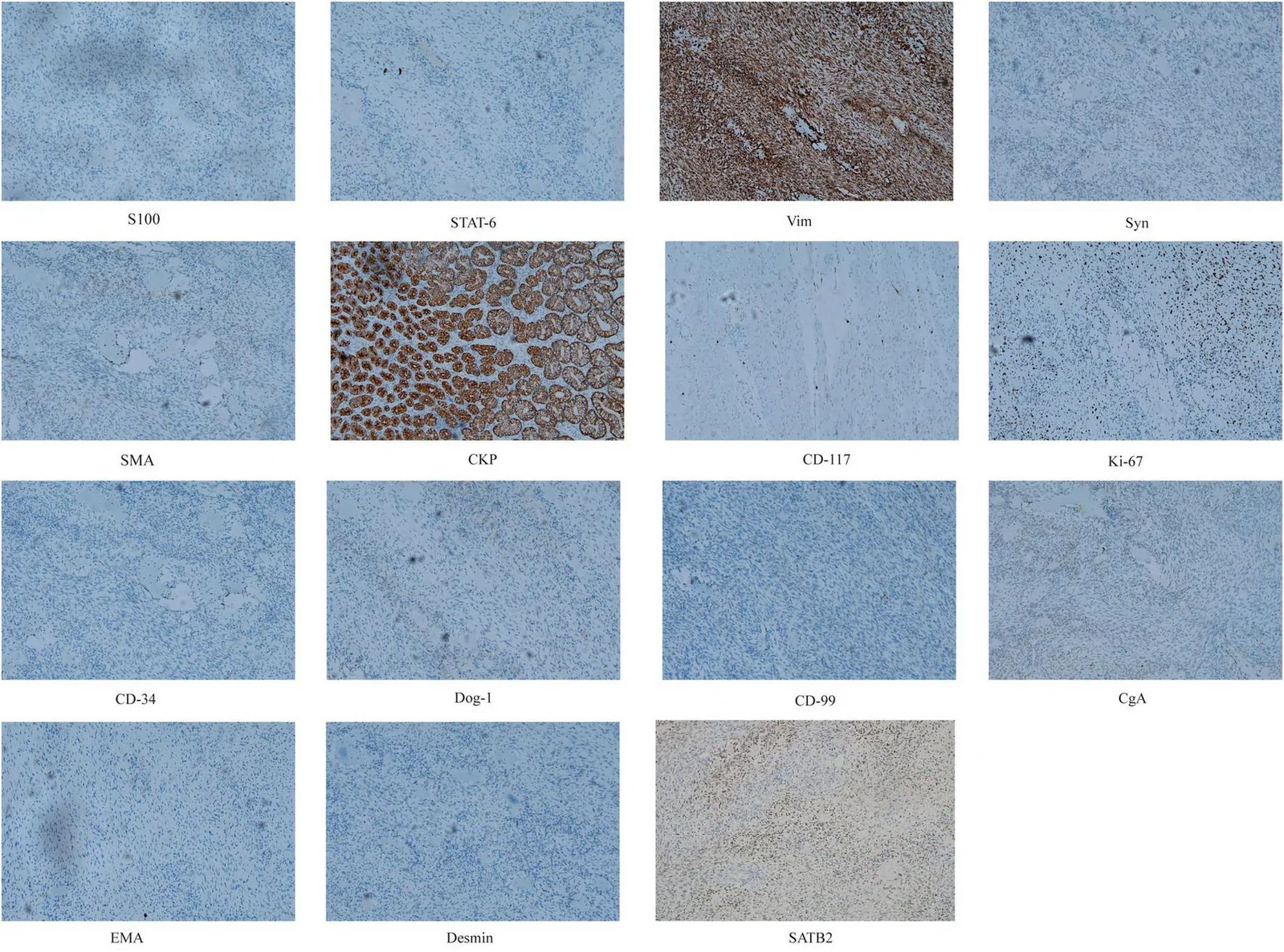
Immunohistochemical results (10×).

**FIGURE 3:**
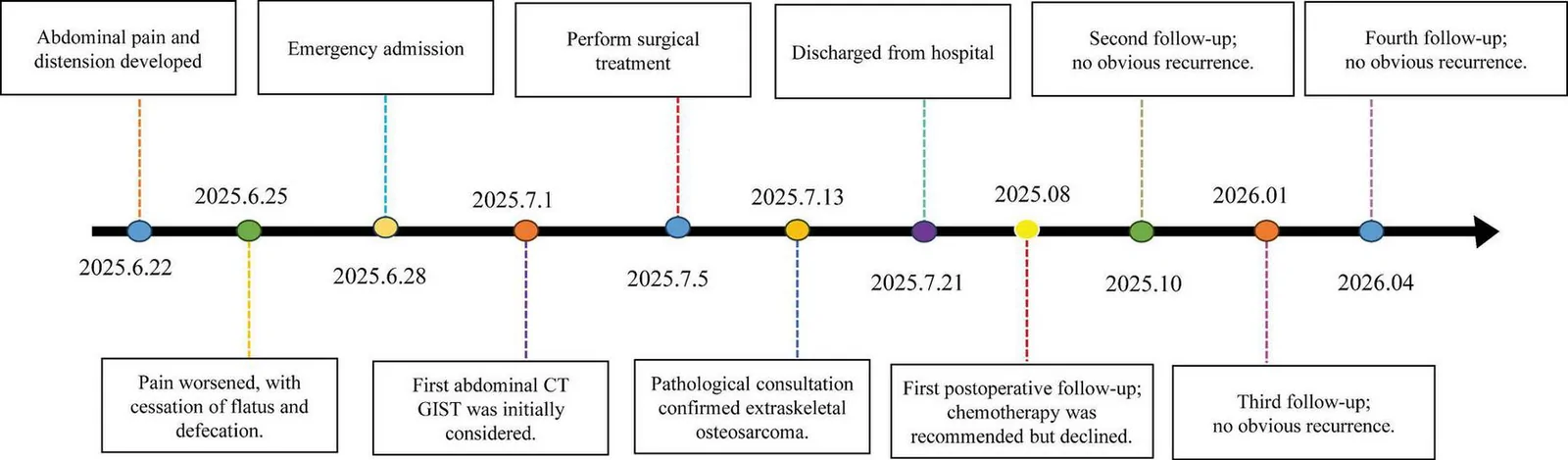
Timeline with relevant data about the onset, diagnosis, and therapy of the patient with primary gastric osteosarcoma.

Because of progressive worsening of symptoms, the patient underwent emergency distal gastrectomy, radical resection of the intra-abdominal mass, Billroth I anastomosis, and regional lymph node dissection on July 5, 2025. Gross examination showed a partial gastrectomy specimen measuring 15 cm × 12 cm × 13 cm. A mass was identified within the submucosal and muscular layers of the stomach, measuring 11 cm × 9.5 cm × 13 cm. On the cut surface, a small portion near the submucosa was gray-white and firm, with a calcified texture. The tumor infiltrated into the subserosal layer. Most of the remaining tumor area showed necrosis, with gray-yellow to gray-red cut surfaces and focally ill-defined borders. A small amount of perigastric adipose tissue was present. Microscopically, the tumor was composed of spindle-shaped cells arranged in sheets. Multifocal lace-like irregular osteoid formation and focal osteoid tissue formation were observed in the stroma (Figures 1C, D). The immunohistochemistry results showed S100(−), STAT-6(−), Vim(+), SMA(−), CKP(−), CD117(−), CD34(−), Dog-1(−), CD99(−), EMA(−), Desmin(−), CgA(−), Syn(−), SATB2(+), Ki-67(>50%). All immunohistochemical results can be seen in Figure 2. The patient recovered uneventfully after surgery. Considering the high-grade morphology and large tumor size, adjuvant chemotherapy using a regimen similar to that for conventional osteosarcoma was recommended after multidisciplinary discussion. However, the patient’s family declined further adjuvant treatment because of concerns regarding her poor general condition. At 1, 3, 6, and 9 months after surgery, the patient reported no obvious discomfort. Follow-up contrast-enhanced abdominal CT showed postoperative changes after gastrectomy and a reduction in abdominopelvic fluid compared with the preoperative findings. No radiological evidence of local recurrence or distant metastasis was observed. The patient is scheduled to undergo close surveillance with chest and abdominal CT every 3 months. The timeline of patient’s diagnosis, treatment and follow-up has been displayed in Figure 3. This case report was prepared in accordance with the CARE guidelines and provides a complete checklist as Supplementary material.

## 3 Literature review

We searched CNKI, Wanfang, VIP, PubMed, Web of Science, and Embase using the keywords “extraskeletal osteosarcoma,” “extraosseous osteosarcoma,” “gastric,” “stomach,” “digestive system,” and “gastrointestinal tract.” To date, no previously published case of primary gastric extraskeletal osteosarcoma has been identified. Therefore, the present case may represent the first reported case of primary gastric EOS. We also reviewed previously reported cases of EOS and summarized their imaging features, histological characteristics, treatment strategies, and clinical outcomes in Table 1. Radiologically, most digestive-system EOS lesions showed infiltrative growth with irregular enhancement. Many tumors contained mature osteoid components, and some showed extensive necrosis with irregular calcification. Histologically, EOS usually demonstrates high-grade morphology, including marked cytological atypia, frequent mitotic activity, tumor necrosis, and characteristic irregular lace-like osteoid matrix formation.

**TABLE 1:** Previously reported cases of extraskeletal osteosarcoma.

References	Age	Sex	Location	Imaging findings	Histological findings	Treatment	Outcome
Wegner et al. (15)	45	Male	Esophagus	CT showed a lobulated calcified esophageal mass at the T4 level.	Biopsy showed mitotically active spindle cells with focal osteoid formation.	Esophageal stenting and doxorubicin plus cisplatin chemotherapy for 4 cycles.	Tracheal metastasis; died 8 months later.
Tamang et al. (16)	50	Male	Liver	CT showed a right hepatic lobe mass measuring 4.4 cm × 4.8 cm × 4.8 cm, with extensive necrosis and peripheral enhancement.	Biopsy showed malignant fibroblast-like cells producing lace-like osteoid matrix.	Portal vein embolization.	Died 6 weeks later.
Iannaci et al. (9)	81	Female	Colorectum	Thickening of the rectal wall at the rectosigmoid junction.	Biopsy showed a highly cellular mesenchymal lesion composed of spindle and epithelioid cells with moderate nuclear pleomorphism. Extensive necrosis and hemorrhage were present. The calcified component consisted of extensive osteoid deposition and interconnecting mature bony trabeculae lined by pleomorphic osteoblasts, with osteoclast-like giant cells.	Miles’ operation followed by postoperative doxorubicin plus ifosfamide chemotherapy for 4 cycles.	Died 6 months after surgery due to cardiac and respiratory complications.
Nie et al. (17)	75	Female	Sigmoid mesocolon	MRI showed a large, irregular, multilocular, solid-cystic mass with complex signal intensity in the sigmoid mesocolon, with an indistinct boundary with the uterus.	The tumor consisted of polygonal tumor cells and abundant osteoid. The tumor cells showed marked atypia, high mitotic activity, and atypical mitotic figures. Eosinophilic osteoid matrix was closely intermixed with tumor cells and showed focal deposition.	Surgical resection.	Recurrence at the anastomosis and transverse colon; died 9 months later.
Heukamp et al. (18)	61	Male	Small intestinal mesentery	CT showed a 10 cm × 6 cm × 5 cm mass in the left lower abdomen, with diffuse margins and focal calcification.	Infiltration of osteoblast-like tumor cells with abundant trabecular bone formation.	Surgical resection followed by postoperative chemotherapy: doxorubicin plus cisplatin for 3 cycles, cyclophosphamide plus cisplatin for 3 cycles, and ifosfamide plus doxorubicin for 3 cycles.	Tumor recurrence; died 10 months later.
Jiang et al. (19)	66	Female	Liver	FDG PET/CT showed multiple partially calcified lesions with central necrotic areas.	Tumor cells were distributed in sheets and showed marked pleomorphism and irregular morphology. The tumor cells produced irregular glassy osteoid matrix, and some osteoid matrix showed calcification.	Liver transplantation followed by postoperative chemotherapy.	Recurrence 6 months after surgery; died.
Nojima et al. (20)	23	Male	Jejunum	Not reported.	The tumor was composed of spindle cells arranged in a whorled pattern and irregular nodular masses. Numerous mitotic figures and malignant osteoid tissue were present.	Surgical resection followed by combination chemotherapy with vincristine, methotrexate, doxorubicin, and cyclophosphamide.	Died 19 months after surgery due to exophytic tumor invasion of the heart.
Ratnagiri et al. (21)	46	Not reported.	Retroperitoneum	A tumor arising from the lower pole of the left kidney, infiltrating the descending colon.	Microscopy showed large malignant elongated spindle cells with pleomorphic nuclei arranged in an irregular fascicular pattern.	A left radical nephrectomy with resection of the descending colon *in toto* was performed; six cycles of combined administration of adriamycin and ifosfamide.	Pulmonary metastases 6 months after completion of chemotherapy and succumbed to the disease.
Nithiyanandham et al. (22)	73	Male	Testicle	An ill-defined heterogeneously enhancing mass with extensive amorphous intratumoral calcifications.	Histology revealed spindle to polyhedral cells with scant eosinophilic cytoplasm and hyperchromatic nuclei, with 14 mitoses per 10 high power field. Foci of osteoid formation, calcification, and osteoclast-like multinucleated giant cells were also noted.	Left high inguinal orchidectomy with en bloc hemiscrotectomy.	After 8 months of follow-up, the condition has stabilized and has not recurred.
Nagpal et al. (23)	62	Male	Retroperitoneum	Right-sided large 20 cm × 15 cm heterogeneous retroperitoneal mass with contrast enhancement, which did not envelope any major organ or vessel, and calcification but was invading into the spine.	Fasicles of pleomorphic spindle cells amidst collagenized stroma at places resembling osteoid with interspersed foci which showed multinucleated osteoclast-like giant cells and few hemosiderin-laden macrophages.	Pre-operative radiotherapy; right extended lumbar incision with an en bloc resection of the retroperitoneal mass with partial vertebrectomy and spine fixation.	16 months after diagnosis and treatment, patient has no recurrence.
Manning et al. (24)	73	Male	Parotid gland	Not reported.	Light-optic examination of hematoxylin and eosin stained tissue sections demonstrated three principal components: osteoid, atypical stromal cells and osteoclast-like giant cells	Parotidectomy	Not reported.
Luna-Ortiz et al. (25)	74	Female	Head and neck	CT scan disclosed the presence of a 13 cm × 12 cm tumor that presented a central area of necrosis, which was in direct contact with the carotid artery and the internal jugular vein	Microscopically the tumor was mainly composed of spindle cells arranged in sheaths and trabeculae in which malignant osteoid was laid down by atypical osteoblasts scattered in several areas	Complete tumor resection and a reconstruction with delto-pectoral flap was performed; adjuvant radiotherapy (66.6 cgy) was delivered	Died 3 months after treatment
Liu et al. (26)	64	Female	Knee joint	CT showed nodular high- density shadow in the subcutaneous tissue of the anterior inferior part of the left knee joint. MRI showed a nodular short t2 signal shadow in the subcutaneous tissue of the anterior and inferior part of the left knee, with high signal intensity and low signal edge by fat suppression.	Postoperative pathology revealed extensive irregular proliferation of trabecular bone-like tissue with significant degeneration and necrosis.	Tumor resection biopsy, joint debridement, ligament reconstruction, pedicled fascial flap formation, skin grafting, and neurovascular exploration and decompression. Over the course of 11 cycles, three different chemotherapy regimens were administered.	The patient elected to undergo amputation.
Khanna et al. (27)	67	Male	Penis	Chest CT scan was normal, and no other significant findings were noted on the abdominal/pelvic CT scan.	The pathological findings of the specimen were high-grade sarcoma, with osteoid production.	Partial penectomy	It is in good condition with no signs of recurrence
Ito et al. (28)	67	Female	Kidney	CT scan showed a huge renal tumor with accompanying calcifications	Tumor cells exhibit osteoblastic characteristics and undergo osteoid and bone formation.	Nephrectomy and 2 cycles of cisplatin treatment.	Died 4 months after surgery
Flynn et al. (29)	77	Female	Kidney	There is heterogeneous filling defect in the left renal pelvis, extending to multiple renal calyces, accompanied by irregular dystrophic calcification	Multinucleated osteoclasts are located in the matrix of mitotic spindle-shaped cells, with visible patches of osteoid tissue and necrotic areas.	Radical nephroureterectomy	Alive with no evidence of disease 30 months after surgery
Do et al. (30)	56	Male	Kidney	CT showed a 4 cm × 5 cm × 5 cm heterogeneous lesion at the renal hilum, abutting the left psoas muscle	Histopathology revealed osteoid and chondroid matrix.	Laparoscopic radical nephrectomy	Systemic bone metastasis occurred within 6 months
Dabas et al. (31)	20	Male	Parotid gland	MRI showed a 12 cm × 10 cm × 8 cm lesion centered in the right parotid gland, involving paraspinal muscles, c1-c2 vertebrae and extending into the parapharyngeal space.	Osteoid and chondroid matrix arranged in an atypical formation	Complete tumor resection, followed by adjuvant radiotherapy	The condition remained stable 6 months after surgery
Yahaya and Odida (32)	16	Female	Breast	An ultrasound scan (USS) was done, which revealed a large irregular predominantly solid mass with cystic areas and large egg shaped calcification	The tumor was composed of atypical spindle cells and large pleomorphic epithelioid cells and multinucleated cells of osteoclastic type which were forming a new bone matrix (osteoid)	Extensive excisional biopsy and traditional Chinese medicine treatment.	No recurrence was observed 10 months after surgery
Zhang et al. (33)	64	Female	Ureter	Doppler ultrasound showed right renal atrophy, separation of the left renal pelvis (measuring 1.6 cm) and dilatation of the proximal ureter.	Biopsy showed focal necrosis, marked cellular pleomorphism (including mitotically active spindle cells, scattered multinucleated giant cells and inflammatory cells) and focal areas containing osteoid.	Surgical resection.	No local recurrence or distant metastasis was found at 6 months after surgery.
Wodowski et al. (34)	12	Female	Left sternocleidomastoid muscle	Magnetic resonance imaging of the neck showed an enhancing mass measuring 2 cm in diameter within the left sternocleidomastoid muscle at the level of the third cervical vertebra. Computed tomography of the chest showed multiple noncalcified nodules consistent with metastases in the upper and lower lobes of the right lung.	Multinucleate tumor giant cells and osteoclast-like giant cells, the latter loosely arranged around areas of hemorrhage, were scattered throughout the tumor.	Two cycles of neoadjuvant vincristine, doxorubicin, and ifosfamide for 6 weeks achieved >50% tumor reduction and resolution of pulmonary nodules. Wide local excision was followed by radiotherapy (54 gy to primary site, 18 gy to lungs) and 5 additional cycles of the same regimen.	Absence of disease for 2.8 years since the initial diagnosis.
Wang et al. (7)	52	Male	Intraperitoneal	Abdominal CT demonstrated multiple irregular mixed-density lesions in the left lower abdomen and periaortic region, well-defined margins, patchy and linear calcifications, the largest 5.0 cm × 6.3 cm, with heterogeneous contrast enhancement.	Spindle cell tumors were accompanied by a large number of hyaline degeneration and small focal ossification	Primary tumor resection was performed, followed by recurrence at 2 years, and subsequent palliative surgery.	Died 2 months after the second operation.
Vanhooteghem and Theate (35)	66	Female	Right thigh scar	Extensive total body radiologic workup revealed absence of bone lesions in any body site.	Histopathologic features were characterized by predominant chondroid matrix with marked cellularity, high-grade atypia, and high mitotic activity.	The patient rejected the therapeutic proposal of definitive large surgical excision	Not reported.
Vaquero et al. (36)	63	Female	Inner side of right thigh	Radiographs revealed a well-defined 4 cm calcified shadow at the middle third of the right thigh, separate from the underlying bone.	Peripheral mature osseous trabeculae surrounding central osteoid; pleomorphic hyperchromatic polygonal cells with large nuclei and frequent atypical mitoses; admixed with multinucleated epulis-type giant cells.	Underwent chemotherapy	Not reported.
Hertel et al. (37)	69	Female	Thyroid	Neck MRI showed a 6 cm × 10 cm right paralaryngeal mass with severe tracheal displacement and stenosis, contiguous with the right thyroid lobe.	Predominant spindle cell sarcoma without epithelial differentiation; focal chondroid and osseous differentiation; sparse residual thyroid follicles, fibrotic adenomas and colloid nodules.	Bilateral total thyroidectomy combined with palliative tumor debulking toward larynx and hypopharynx, plus tracheotomy	Died of progressive tumor disease at 4 months post-diagnosis.
Skensved and Søndergaard (38)	56	Male	Right renal fossa	Abdominal ultrasound showed a well-demarcated mass with multiple confluent echo-free cystic cavities, and an ill-defined enlarged right kidney with its lower pole extending 1–2 cm below the umbilicus.	Consistent osteosarcoma morphology with variable differentiation: well-differentiated areas with mature osteoid trabeculae and rare mitoses; poorly differentiated areas with dense spindle cells and up to 10 mitoses/10 hpf. Scattered multinucleated giant cells, scarce chondroid, massive central necrosis with viable tumor mainly at the periphery.	Underwent radical resection of retroperitoneal primary tumor, right kidney and proximal right ureter, followed by staged metastasectomy.	Died 3 months after the last operation
Shringarpure et al. (39)	35	Male	The base of the penis	Coronal CT scan of pelvis showing extensive ossification in the corpora cavernosa of the penis	Spindle-shaped cells with anaplasia and areas of osteoid formation were observed.	Total penectomy with urinary diversion, followed by three cycles of adjuvant chemotherapy.	No recurrence at 1-year follow-up.
Schiller et al. (40)	70	Female	Intradural-extramedullary spinal	Lumbar MRI showed a diffuse intradural mass from t12 to sacrum with heterogeneous signal and homogeneous enhancement, as well as multiple oval hyperintense lesions in the lower spinal canal.	Hypercellular spindle cell stroma with nuclear atypia, variable nuclear size, rare mitoses, and abundant immature lace-like osteoid matrix.	L2/l3 laminectomy with durotomy, tumor debulking, and nerve root rhizolysis for spinal decompression; subtotal resection due to dense adhesion to nerve roots.	Died 6 weeks after surgery.
Karfis et al. (41)	58	Female	Lung	Contrast-enhanced chest CT showed a 2.1 cm heterogeneous calcified mass with malignant features in the left lingula.	Malignant osteoid matrix produced by neoplastic spindle cells, with coexisting osteoblastic and chondroblastic areas, and widespread sclerosis.	Radical resection of the left upper lobe of the lung, followed by standard chemotherapy and radiotherapy.	Alive at 6 months with no recurrence or metastasis on imaging.

The prognosis of EOS is generally poor. Most reported cases of digestive-system EOS were treated primarily by surgical resection, with the aim of achieving R0 resection whenever feasible. However, the benefits of adjuvant chemotherapy or radiotherapy remain uncertain because of the limited number of cases and the lack of standardized treatment guidelines. Reported outcomes suggest a high risk of recurrence and low long-term survival. The present case provides an additional reference for clinicians encountering similar rare gastric tumors and contributes to the limited literature on EOS involving the digestive system.

## 4 Discussion

We report a rare case of primary gastric extraskeletal osteosarcoma. To our knowledge, only a few cases of EOS originating from the small intestine, colon, or mesentery have been reported in the literature (9). A comprehensive search of both Chinese and English databases identified no previously published case of primary gastric EOS. Therefore, the present case may represent the first reported case of EOS arising primarily from the stomach. This case broadens the known anatomical spectrum of EOS and highlights the diagnostic challenges associated with this rare malignancy.

The initial radiological findings in this patient mimicked those of more common gastric or intra-abdominal tumors, particularly gastrointestinal stromal tumors. The patient mainly presented with obstructive symptoms, including abdominal pain, abdominal distension, cessation of flatus and defecation, nausea, and vomiting. These manifestations were non-specific and did not allow a definite preoperative diagnosis. In this case, the final diagnosis depended on histopathological identification of malignant osteoid formation, supported by immunohistochemical exclusion of other differential diagnoses.

The diagnosis of primary gastric EOS requires careful exclusion of other tumors. Gastrointestinal stromal tumor should be considered first in a large gastric mass with heterogeneous enhancement, necrosis, and calcification; however, the tumor cells in this case were negative for CD117 and DOG1. Sarcomatoid carcinoma and carcinosarcoma may show spindle-cell morphology, but the tumor cells were negative for cytokeratin-pan and EMA. CKP staining was observed in residual gastric glandular epithelium rather than in tumor cells, supporting its interpretation as an internal epithelial control. Leiomyosarcoma was unlikely because SMA and desmin were negative. Malignant peripheral nerve sheath tumor was not supported because S100 was negative. Solitary fibrous tumor, Ewing sarcoma/primitive neuroectodermal tumor, and neuroendocrine neoplasm were also excluded based on negative STAT6, CD99, chromogranin A, and synaptophysin staining. In addition, the tumor showed direct formation of malignant lace-like osteoid matrix, which is the key histological feature of EOS. After multidisciplinary discussion and exclusion of metastatic skeletal osteosarcoma, the diagnosis of primary gastric EOS was established.

Because no standard treatment strategy has been established for primary gastric EOS, management should be individualized. In the present case, emergency surgery was performed because of progressive obstructive symptoms, and R0 resection was achieved. Complete surgical resection remains the cornerstone of treatment for localized EOS and may provide the best opportunity for long-term disease control. However, the role of adjuvant chemotherapy remains controversial. Most chemotherapy regimens reported in the literature are extrapolated from conventional skeletal osteosarcoma, but their efficacy in EOS is inconsistent. Some studies have suggested a survival benefit from chemotherapy, whereas others have found no significant improvement in survival (8, 10–12). Considering the large tumor size, high-grade morphology, and aggressive biological behavior of EOS, adjuvant chemotherapy was recommended after multidisciplinary discussion in this patient. However, further treatment was declined by the patient’s family because of concerns regarding her general condition.

Given the lack of prognostic data for gastric EOS, the follow-up strategy in this case was based on known prognostic factors for EOS at other anatomical sites. Reported adverse prognostic factors include tumor size greater than 5 cm, high-grade histology, positive surgical margins, and distant metastasis. Although the tumor in this case was large and high grade, the patient underwent R0 resection and had no evidence of lymph node or distant metastasis, which may indicate a relatively favorable short-term outcome. Nevertheless, EOS has a high risk of recurrence and metastasis, especially pulmonary metastasis. Therefore, close surveillance with chest and abdominal CT every 3 months was planned. We also plan to monitor serum alkaline phosphatase and lactate dehydrogenase levels during follow-up, as these markers are clinically relevant in osteosarcoma surveillance (13, 14). In this patient, preoperative chest CT suggested old pulmonary tuberculosis. Although there is currently no direct evidence that old pulmonary tuberculosis increases the risk of pulmonary metastasis in EOS, careful chest imaging follow-up is particularly important because the lung is a common metastatic site for osteosarcoma.

This report has several limitations. First, it describes a single case, and therefore, no definitive conclusions can be drawn regarding the biological behavior or optimal management of primary gastric EOS. Second, the follow-up period remains relatively short, and long-term outcomes require continued observation. Third, molecular testing was not performed, which limits further exploration of the molecular characteristics and potential therapeutic targets of this tumor. Future accumulation of similar cases through multicenter collaboration or international registries will be necessary to better define the clinicopathological features, prognostic factors, and treatment strategies for EOS. Molecular profiling may also help clarify tumor biology and identify potential roles for targeted therapy or immunotherapy.

In conclusion, primary gastric EOS is an extremely rare malignancy that should be considered in the differential diagnosis of atypical gastric tumors with calcification, necrosis, and spindle-cell morphology. Accurate diagnosis requires adequate sampling, careful histopathological evaluation, and systematic immunohistochemical exclusion of mimicking tumors. Complete surgical resection remains the main treatment for localized disease, while the role of adjuvant chemotherapy should be determined on an individual risk-benefit basis. Close long-term follow-up is essential because of the high risk of recurrence and metastasis.

## 5 Patients’ perspective

The patient stated: “Before admission, I was very worried because my abdominal pain and distension became increasingly severe. After the medical team explained my condition, emergency surgery was performed promptly. My symptoms improved after the operation, and I recovered well. Although my family declined further chemotherapy because of concerns about my physical condition, I understand the need for close follow-up. At present, I feel well and have no obvious discomfort.”

## Data Availability

The raw data supporting the conclusions of this article will be made available by the authors, without undue reservation.
